# Cross-Evaluation of Reflectivity from NEXRAD and Global Precipitation Mission during Extreme Weather Events

**DOI:** 10.3390/s22155773

**Published:** 2022-08-02

**Authors:** Melisa Acosta-Coll, Abel Morales, Ronald Zamora-Musa, Shariq Aziz Butt

**Affiliations:** 1 Department of Computer Science and Electronic, Universidad de la Costa, Barranquilla 080002, Colombia; 2 Department of Electrical Engineering, University of Puerto Rico at Mayagüez, Mayagüez, PR 00681-9018, USA; abel.morales@upr.edu; 3 Department of Industrial Engineering, Universidad Cooperativa de Colombia UCC, Barrancabermeja 687031, Colombia; ronald.zamora@campusucc.edu.co; 4 Department of Computer Science, The University of Lahore, Lahore 54000, Pakistan; shariq2315@gmail.com

**Keywords:** cross-evaluation, reflectivity, NEXRAD, GPM, hurricane, ground validation system, ground radar

## Abstract

During extreme events such as tropical cyclones, the precision of sensors used to sample the meteorological data is vital to feed weather and climate models for storm path forecasting, quantitative precipitation estimation, and other atmospheric parameters. For this reason, periodic data comparison between several sensors used to monitor these phenomena such as ground-based and satellite instruments, must maintain a high degree of correlation in order to issue alerts with an accuracy that allows for timely decision making. This study presents a cross-evaluation of the radar reflectivity from the dual-frequency precipitation radar (DPR) onboard the Global Precipitation Measurement Mission (GPM) and the U.S. National Weather Service (NWS) Next-Generation Radar (NEXRAD) ground-based instrument located in the Caribbean island of Puerto Rico, USA, to determine the correlation degree between these two sensors’ measurements during extreme weather events and normal precipitation events during 2015–2019. GPM at Ku-band and Ka-band and NEXRAD at S-band overlapping scanning regions data of normal precipitation events during 2015–2019, and the spiral rain bands of four extreme weather events, Irma (Category 5 Hurricane), Beryl (Tropical Storm), Dorian (Category 1 hurricane), and Karen (Tropical Storm), were processed using the GPM Ground Validation System (GVS). In both cases, data were classified and analyzed statistically, paying particular attention to variables such as elevation angle mode and precipitation type (stratiform and convective). Given that ground-based radar (GR) has better spatial and temporal resolution, the NEXRAD was used as ground-truth. The results revealed that the correlation coefficient between the data of both instruments during the analyzed extreme weather events was moderate to low; for normal precipitation events, the correlation is lower than that of studies that compared GPM and NEXRAD reflectivity located in other regions of the USA. Only Tropical Storm Karen obtained similar results to other comparative studies in terms of the correlation coefficient. Furthermore, the GR elevation angle and precipitation type have a substantial impact on how well the rain reflectivity correlates between the two sensors. It was found that the Ku-band channel possesses the least bias and variability when compared to the NEXRAD instrument’s reflectivity and should therefore be considered more reliable for future tropical storm tracking and tropical region precipitation estimates in regions with no NEXRAD coverage.

## 1. Introduction

Hurricanes, or tropical cyclones (TC), are characterized by high-speed winds, heavy precipitation, and low atmospheric pressure, that transform into natural disasters as they reach land [1]. The major devastation occurs as a result of flooding [2,3]; therefore, rainfall estimation is very important for emergency evacuation planning. When a hurricane makes landfall, the most intense precipitation tends to occur in the vicinity of coastlines; predicting this event is a significant operational challenge [4,5]. However, flooding due to precipitation is not limited to coastlines, as seen in recent hurricanes where deadly floods reached well inland.

The storm’s progression and resulting hazard effects on land are often highly uncertain. Since the ensemble of forecasts changes during a TC, the uncertainty becomes dynamic, and it only ends when the storm’s evolution is known completely [6,7]. In order to generate a timely early warning system before and after the severe precipitation event, it is necessary to have instruments with high accuracy for detection, measurement, and tracking of storms [8].

A powerful instrument to monitor severe events such as tropical cyclones is the Global Precipitation Measurement (GPM) mission, an international network of satellites used to provide accurate and timely information. GPM is an international partnership sponsored by NASA and the Japan Aerospace Exploration Agency (JAXA) that launched on 27 February 2014 [9]. This network can provide valuable information needed to monitor the evolution of devastating storms, and helps scientists study their fast-moving and rapidly evolving nature [10]. GPM carries two instruments: a passive microwave radiometer GMI and a dual-frequency precipitation radar (DPR) [11]. The DPR consists of Ku-band (KuPR) and Ka-band (KaPR) radars on the GPM spacecraft bus, which are capable of measuring precipitation simultaneously [12]. These radars operate at frequencies of 13.91 GHz and 35.56 GHz, respectively, and provide a three-dimensional observation of rain with an accurate estimation of rainfall rate. They are co-aligned and provide the same footprint location on the earth of 5 km. KuPR is suitable for heavy rainfall in the tropical region, and KaPR suitable for light rainfall in the higher latitude region [13]. DPR lower and upper thresholds for rain rate measurements are 0.22 and 110.00 mm/h, respectively [14].

Ground-based weather radars, which provide a spatial resolution of 1 km or less, are also used worldwide to detect and analyze rapidly moving severe storms, and to send timely alerts to the community [15]. However, during severe and hazardous weather events, these instruments can be damaged and consequently stop providing valid information; this occurred when Hurricane Maria made landfall in Puerto Rico in 2017 and destroyed the only NEXRAD on the island. Therefore, during severe weather events, it is vital to have redundancy provided by satellite instruments in order to detect and monitor the events, while ensuring the uninterrupted transmission of timely information [16].

When there are several instruments monitoring a weather event in the same region, the information must be consistent between the instruments, especially for large areas where hydrologic applications need information from multiple radar data. This information is susceptible to radar measurement differences in the overlapping zones, due to radar calibration, range effect, or both [16]. In order to mitigate this problem, NASA developed an algorithm to match reflectivity from DPR and NEXRAD over different sampling volumes, and this effort has been of great importance for evaluating and improving algorithm performance [17].

One of the most affected U.S. territories during hurricane season in the Atlantic Ocean is the island of Puerto Rico [18,19]. The most devastating hurricane that has impacted the island was Hurricane Maria in September 2017; however, Hurricane Irma also impacted Puerto Rico during that same month only 10 days earlier. Hurricane Irma was a category 5 hurricane with approximately 175-mile-per-hour winds, and was the strongest observed in the Atlantic in terms of maximum sustained wind [20]. It lasted as a hurricane from 31 August until 11 September, and skirted the northeast region of Puerto Rico on 6 September 2017. This hurricane left more than 1 million people without electricity, some regions without potable water, and damaged roads and communication system infrastructure in Puerto Rico [21].

During Hurricane Irma, the NWS in Puerto Rico used weather satellites and a NEXRAD radar to monitor the severe weather conditions. This radar is located in Cayey (18.12° N, 66.08° W, 886.63 m elevation), is identified as TJUA, and operates at a frequency of 2.7 GHz (S-Band). It has a maximum horizontal range of 462.5 km, and scans the entire island every 6 min with a spatial resolution of 1 km [22].

In 2018, the remnants of Tropical Storm Beryl affected Puerto Rico and the U.S. Virgin Islands on 9 July. Strong winds and heavy rainfall affected Puerto Rico, where the average rainfall ranged from 1 to 6 inches. Several locations reported flash flooding. As a consequence of this tropical storm, at least 24,000 homes and businesses were without electricity, there were several fallen trees, and rivers rose over their banks; however, no injuries were reported [1].

In September 2019, two extreme weather events hit Puerto Rico, Hurricane Dorian in 6 September, and Tropical Storm Karen in 24–25 September. Dorian was the first major hurricane of the 2019 Atlantic hurricane season. Although Dorian was less powerful than Hurricanes Irma and Maria, people from Puerto Rico prepared for the worst since they were still recovering from Maria. Fortunately, Hurricane Dorian’s projected path unexpectedly swerved northward and left only some residents without electricity, and some areas flooded.

Tropical Storm Karen became downgraded to a tropical depression when it hit Puerto Rico. On its way through the island, flooding occurred and power outages affected less than 10% of the total population.

This study presents an evaluation of GPM-DPR rainfall reflectivity against NEXRAD TJUA radar reflectivity during four extreme weather events and during normal precipitation events, in order to determine the degree of correlation between these two instruments. Measurement data from the extreme weather events in 2015–2019 were statistically analyzed, and reflectivity differences were broken down by precipitation type (stratiform and convective) and radar elevation angle, comparing KaPR and KuPR with NEXRAD TJUA separately.

The study results identified that the correlation coefficient between the data of both instruments during the extreme weather events was moderate to low, and for normal precipitation events the correlation is lower than that for other studies that compared GPM and NEXRAD reflectivity located at other sites in the USA.

However, Tropical Storm Karen had a better correlation coefficient for its four angles compared to the other extreme weather events. Likewise, the ground radar elevation angle and precipitation type have a substantial impact on how well the reflectivities match, and Ku-band possesses the least bias and variability when compared to ground radar reflectivity.

Since extreme weather events are frequent in this area, it highlights the importance of periodically conducting comparative studies to ensure consistency between instruments, in order to provide high accuracy information that allows timely decision making.

The structure of this article is as follows: Section 2 presents a literature review of studies that compare matched data from satellite-based radars and ground radars in different regions of the globe. Section 3 describes the methodology, data, and procedures used to carry out the cross-evaluation. Then, Section 4 presents the results and the discussion of the cross-evaluation. Finally, Section 5 shows the conclusions of this research.

## 2. Literature Review

There have been multiple studies that compared that matched data between satellite-based radars and ground radars in different regions of the globe. The study developed by [23] used space-borne precipitation radar information to quantitatively calibrate ground-based weather radar networks across China. Likewise, researchers from Colorado State University performed ground validation of GPM-DPR observations using an S-band NEXRAD over the Dallas Fort Worth region in Texas, and reported that the reflectivities were well matched. The intercomparison of reflectivity measurements between GPM-DPR and NEXRAD radars carried out by researchers from NASA [24] found that taking samples with narrow temporal gaps helps to reduce sample variability. Likewise, in order to reduce the reflectivity differences among GRs in a similar environment, they suggest applying a bias correction against the DPR. However, more studies are necessary in tropical regions, and it is also necessary to identify possible beam blockages that can affect patterns in the GR intercomparison results from before.

K. R. Morris and M. R. Schwaller from NASA performed a study of the sensitivity of PR-GR measurements for constraints such as range from GR, minimum reflectivity threshold, PR-GR time differences, and other variables. They found that there is a significant difference between PR and GR reflectivities in convective cases, particularly in convective samples from the lower part of the atmosphere [25].

These studies have been deployed all over the world; nevertheless, there are relatively few that have been done for Latin America, especially the Caribbean. I. Arias and V. Chandrasekar performed a cross-validation of GPM with three GR radars from Colombia; two C-band weather radars close to Bogota DC; and another one in San Andres Island (Caribbean Ocean). The results showed that the Colombian radar and GPM observations have a high correlation within 90%, and bias within 1 dBZ [26].

## 3. Methodology

In order to obtain the matched data between GPM-DPR and NEXRAD during four extreme weather events and during normal precipitation events, the data products available from the GPM ground validation system (GVS) validation network (VN) were used.

The VN performs a direct match-up of DPR and GR data using the geometry-matching algorithm developed by NASA from the GPM terrestrial validation system (GVS) [27].

**Figure 1:**
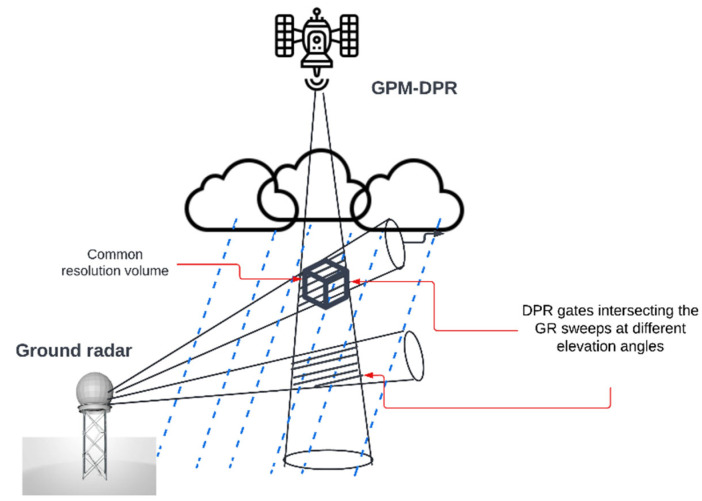
GPM-DPR and ground radar geometric matching.

The algorithm determines the intersection of individual DPR rays with each of the elevation sweeps of the circular scanning ground-based radar, and the data outputs are stored as netCDF files. Due to the randomness of the beam-to-sweep intersections, the horizontal and vertical locations as well as the number of data points in the geometry matching technique are different; moreover, this algorithm allows for the identification of biases between ground observations and satellite recoveries. Figure 1 shows the geometric intersections of DPR gates and GR sweeps at two different elevation angles.

The VN match-up data sets begins on 4 March 2014 (GMI) and 8 March 2014 (DPR, Ka, Ku, DPRGMI), but the matched data with NEXRAD TJUA began in 2015.

In order to select the match-ups, only those gates at or above a specified rain rate or reflectivity threshold are included in the DPR and GR gate averages (variables DPR_dBZ_min, GR_dBZ_min, and rain_min). These results are stored in netCDF variables [9].

NEXRAD TJUA data and GPM Ku-band and Ka-band data for 2015 to 2019, in addition to four extreme weather events that occurred in this same period of time, are compared in terms of reflectivity differences for the first four matching elevation angles for the three scanning modes for the GR, and categorized by precipitation type.

The events for typical cases and for included extreme weather events cases do not surpass the DPR upper threshold sensitivity rain rate of 110.00 mm/h. On average, crossmatching between DPR and GR over NEXRAD TJUA occurs every four days; occasionally, there can be two consecutive days with match data, and up to a week for a match to occur. The average matching duration for GR and DPR is around 40 s, and DPR produces a swath scan every 300 milliseconds. For this reason, DPR is not a good substitute for GR in terms of continuous local weather monitoring; however, it is a useful instrument for GR data calibration and validation, and is also useful in the absence of local GR, as was the case in Puerto Rico after the damages suffered during Hurricane Maria.

**Figure 2:**
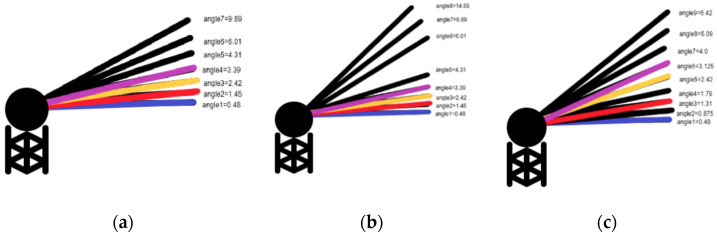
NEXRAD elevation angle scanning modes of operation for (**a**) seven elevations, (**b**) eight elevations and (**c**) nine elevations.

GR has multiple scanning modes with different elevation angles, as Figure 2 shows. Between 2015 and 2019, 165 cases with sufficient precipitation were selected for analysis, as well as the four extreme weather events. Table 1 shows the selected elevation angles and their corresponding beam heights.

The algorithm for the files used is V05A version 1.3, and data within 100 km of the GR are used with a minimum threshold of 15 dBZ and a 7-km distance away from the GR.

Each elevation angle is subcategorized by precipitation type, stratiform and convective; then, the bias is calculated, in addition to the variance, mean absolute error (MAE), mean square error (MSE), and root mean square (RMS), in order to determine variability in reflectivity differences under the different categorizations and subcategorizations, number of samples, and Pearson correlation coefficients (CC).

### 3.1. Extreme Weather Events

#### 3.1.1. Hurricane Irma Data

Hurricane Irma’s eye passed north of Puerto Rico on 6 September by 8 p.m. as a category 5 storm. By 4 a.m. on 7 September, it passed north of the Dominican Republic; consequently, this is a single event comparison between NEXRAD and GPM on 7 September 2017.

**Figure 3:**
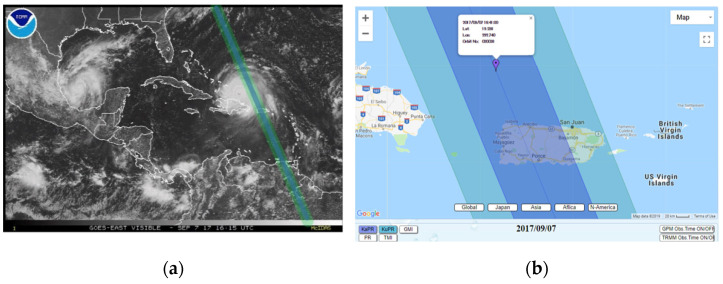
(**a**) GOES East satellite (7 September 2017); (**b**) map of Puerto Rico (7 September 2017).

Figure 3a presents GOES East satellite image of the Caribbean at the moment when Irma and GPM passed over PR on 7 September 2017; Figure 3b shows the map of Puerto Rico with the ascending orbit of GPM over PR on 7 September 2017.

#### 3.1.2. Tropical Storm Beryl

**Figure 4:**
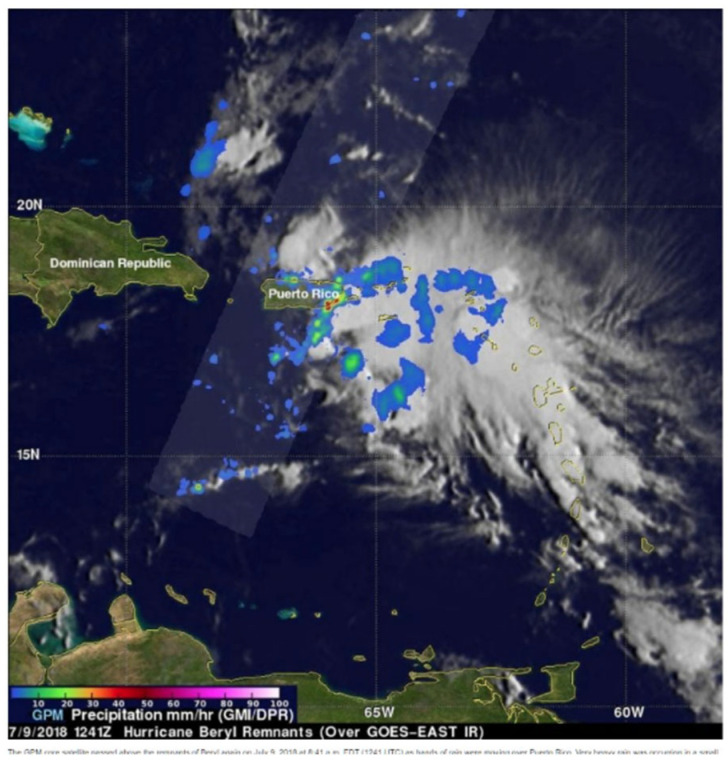
Tropical Storm Beryl over Puerto Rico [28].

Hurricane Beryl weakened to a tropical storm on Saturday, 7 July 2018 as it approached islands in the eastern Caribbean. In Puerto Rico, between 9 and 10 July strong winds were reported; moreover, up to 8 inches of rain fell in some areas. Figure 4 shows Tropical Storm Beryl over Puerto Rico.

#### 3.1.3. Hurricane Dorian

**Figure 5:**
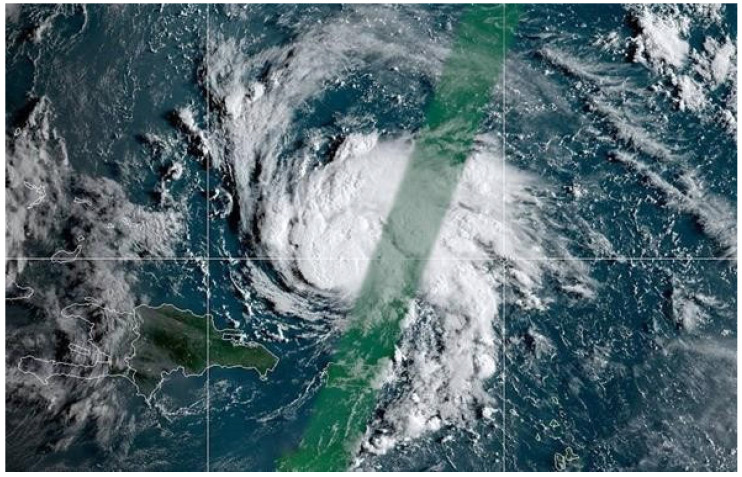
Hurricane Dorian over Puerto Rico [29].

In Puerto Rico, along the east and southeast, between the 28th and 29th of August, Hurricane Dorian left rainfall accumulations of between 4 and 6 inches, and generated flash flooding especially across the eastern end of Puerto Rico. Figure 5 shows the closest point between GPM and GR on 29 August at 7:01 pm local time (11:01 UTC).

#### 3.1.4. Tropical Storm Karen

**Figure 6:**
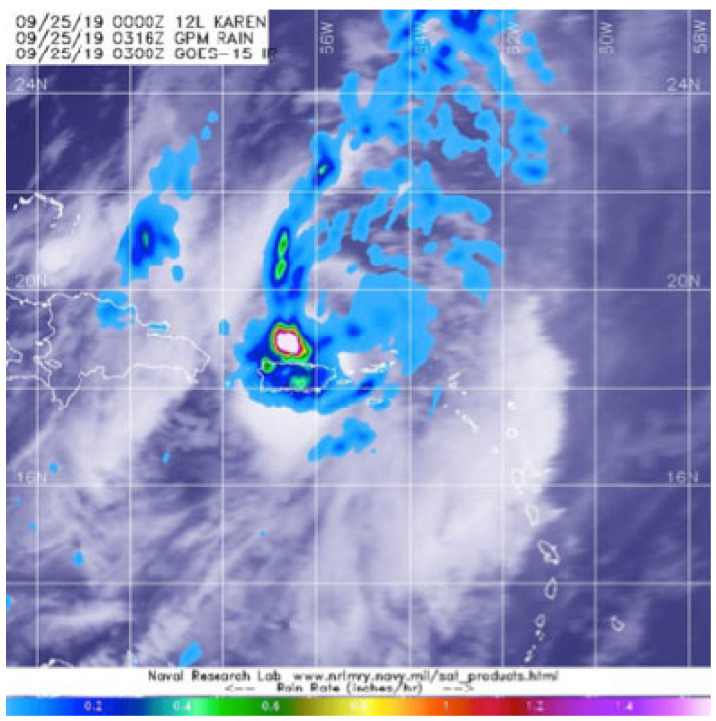
Tropical Storm Karen [30].

Tropical Storm Karen is the weakest event compared to the other three. Figure 6 shows the image captured by the GPM’s core satellite when it passed over Tropical Storm Karen on 25 September 2019 at 11:16 p.m. The most significant damages were heavy rains that led to flooded roads, flash flood warnings, and hazardous marine conditions.

The cross-evaluation of the four extreme weather events (Irma, Beryl, Dorian, and Karen) follow the same categorization and analysis as the normal weather conditions cases from the previous section; the biases were obtained, along with variances, mean absolute errors (MAE), mean square errors (MSE), root mean square (RMS), and the correlation coefficients for each GR elevation angle and subcategorized by precipitation type.

## 4. Results and Discussion

Data were analyzed and classified into normal weather conditions, which were the data for 2015–2019 along with the four included extreme weather cases. Likewise, the results were subcategorized by precipitation type for both cases, and calculated for bias, variance, mean absolute error (MAE), mean square error (MSE), root mean square (RMS), and the correlation coefficient between KuPR vs. NEXRAD TJUA and between KaPR vs. NEXRAD TJUA. 

### 4.1. Normal Weather Conditions

Table 2 shows the statistical results for normal weather conditions.

#### 4.1.1. Angle 1 (0.4843°)

**Figure 7:**
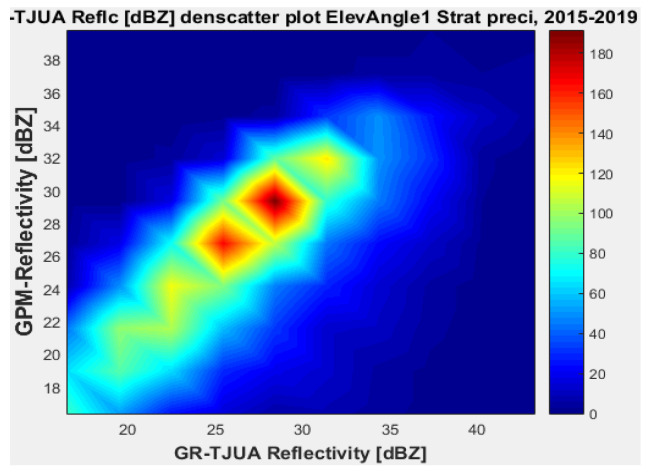
GPM-Ka vs. NEXRAD TJUA for stratiform precipitation (angle 1).

**Figure 8:**
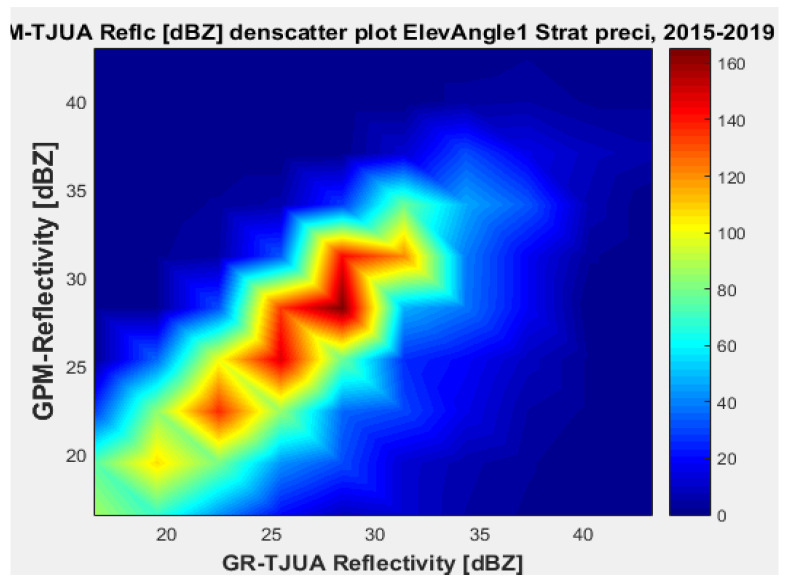
GPM-Ku vs. NEXRAD TJUA for stratiform precipitation (angle 1).

**Figure 9:**
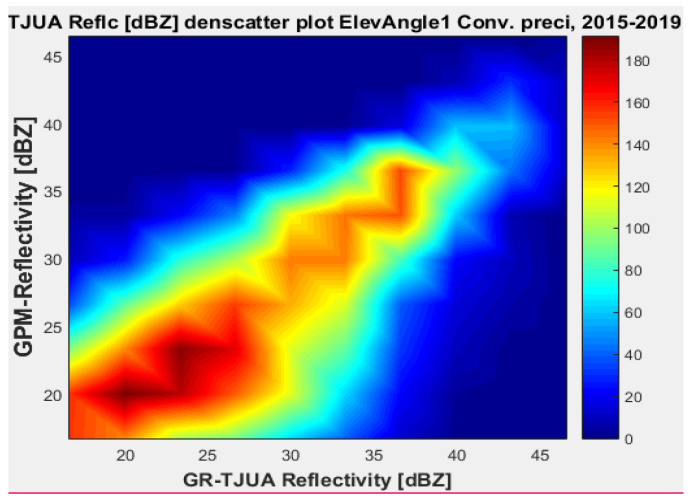
GPM-Ka vs. NEXRAD TJUA for convective precipitation (angle 1).

**Figure 10:**
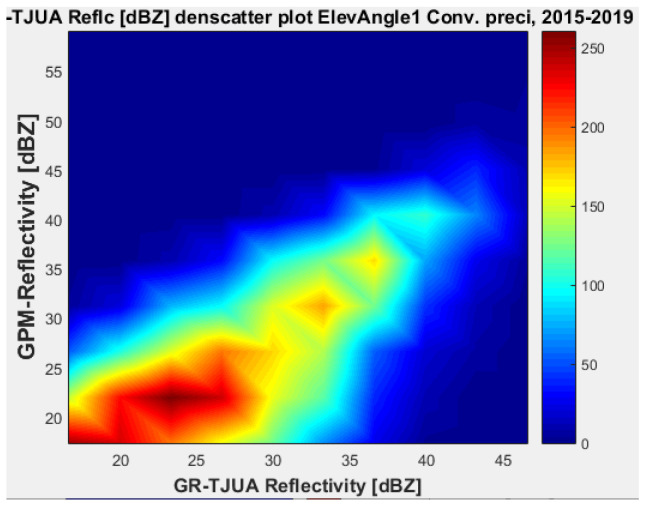
GPM-Ku vs. NEXRAD TJUA for convective precipitation (angle 1).

Figure 7, Figure 8, Figure 9 and Figure 10 represent the scatter density plots for this case for GR angle 1 elevation and the precipitation type.

According to the statistical results for GR elevation angle 1 (0.4843°) for normal weather conditions, 77.5% of the samples correspond to convective precipitation, 22.5% correspond to stratiform, and around 0.16% of the samples are categorized as other (their precipitation types do not correspond to stratiform or convective). The means for KuPR and KaPR show that there is better matching with GR data during stratiform precipitation. However, the variance from KuPR is slightly more significant than KaPR. For both convective and stratiform precipitation, KuPR has better matching with GR data, as can be compared with the scatter plots of Figure 8 and Figure 10.

#### 4.1.2. Angle 2 (1.45°)

**Figure 11:**
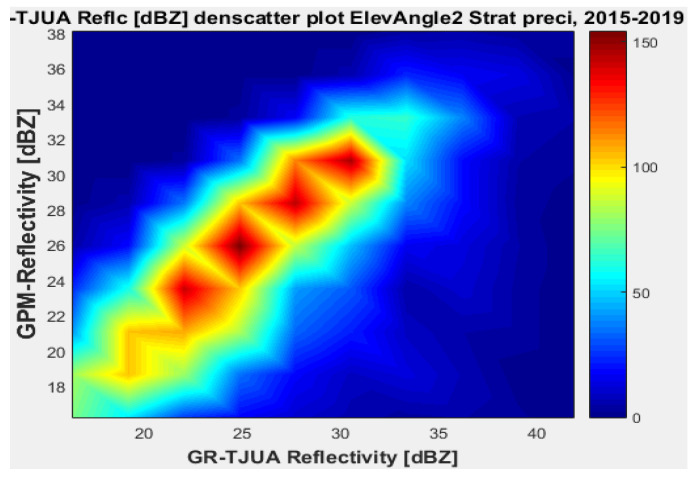
GPM-Ka vs. NEXRAD TJUA for stratiform precipitation (angle 2).

**Figure 12:**
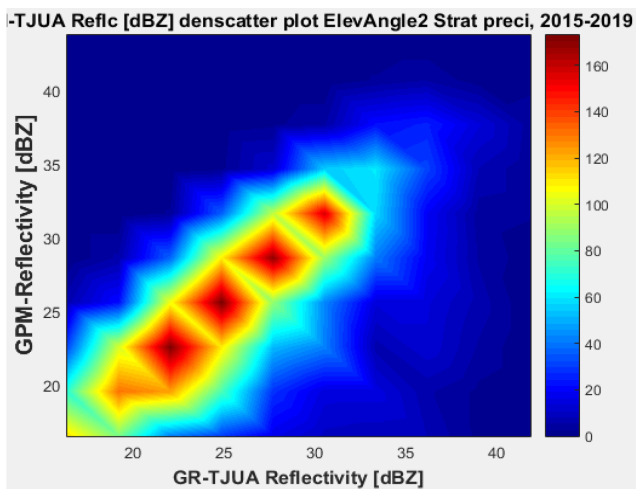
GPM-Ku vs. NEXRAD TJUA for stratiform precipitation (angle 2).

**Figure 13:**
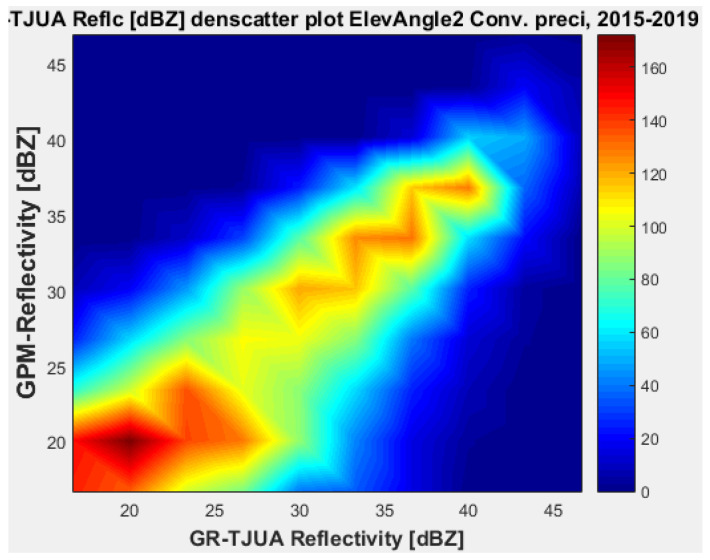
GPM-Ka vs. NEXRAD TJUA for convective precipitation (angle 2).

**Figure 14:**
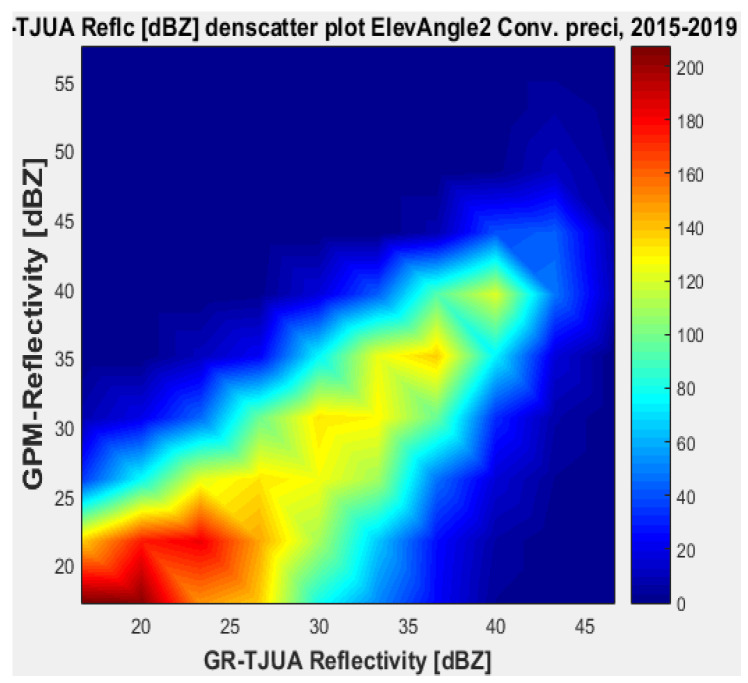
GPM-Ku vs. NEXRAD TJUA for convective precipitation (angle 2).

Figure 11, Figure 12, Figure 13 and Figure 14 represent the scatter density plots for this case for GR angle 2 elevation and the precipitation type.

For angle 2, the composition of the precipitation type is around 30% stratiform, 69.6% convective, and 0.4% classified as other. The mean reflectivity difference for angle 2 has the same behavior as angle 1, where KuPR has better matching for convective and stratiform precipitation, although the mean reflectivity difference is lower for angle 1. Likewise, for angle 2 the KuPR variance is more significant than it is for KaPR. Figure 12 and Figure 14 illustrate that KuPR has better matching for convective and stratiform precipitation.

#### 4.1.3. Angle 3 (2.4219°)

**Figure 15:**
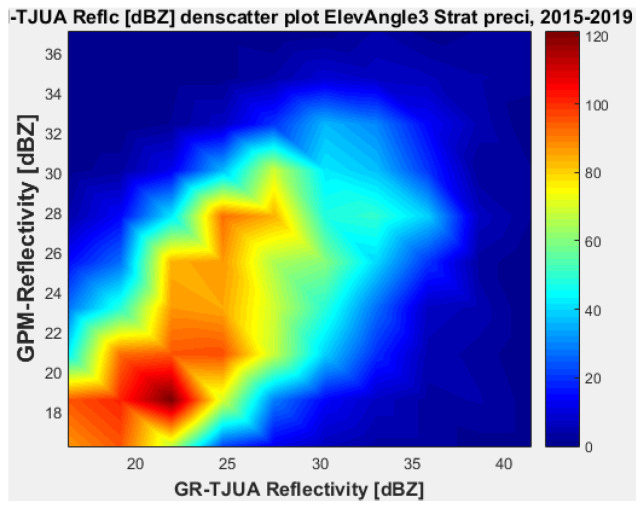
GPM-Ka vs. NEXRAD TJUA for stratiform precipitation (angle 3).

**Figure 16:**
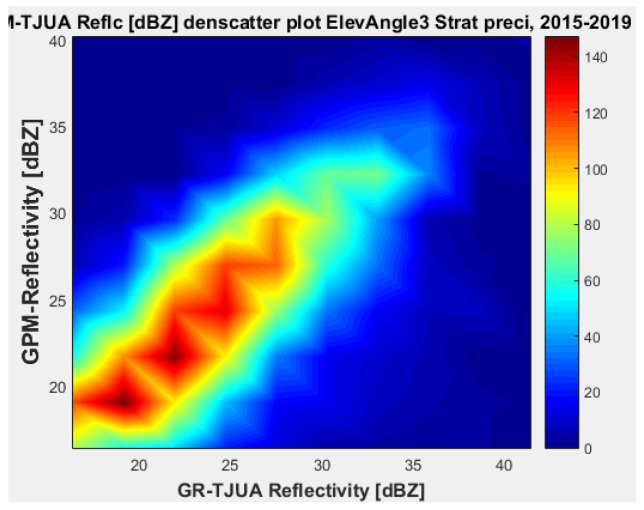
GPM-Ku vs. NEXRAD TJUA for stratiform precipitation (angle 3).

**Figure 17:**
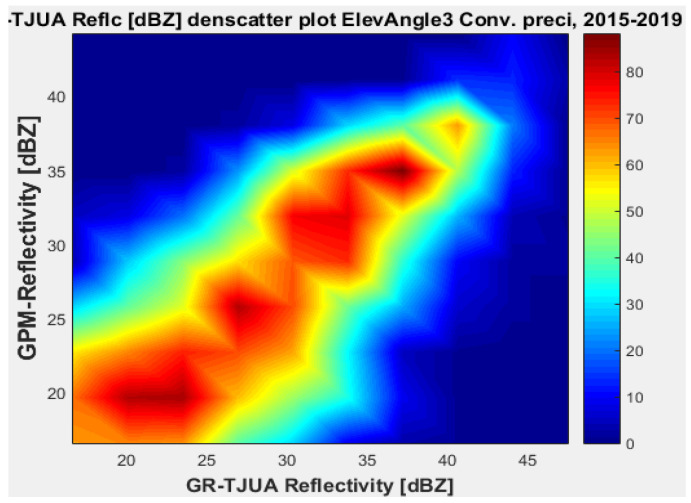
GPM-Ka vs. NEXRAD TJUA for convective precipitation (angle 3).

**Figure 18:**
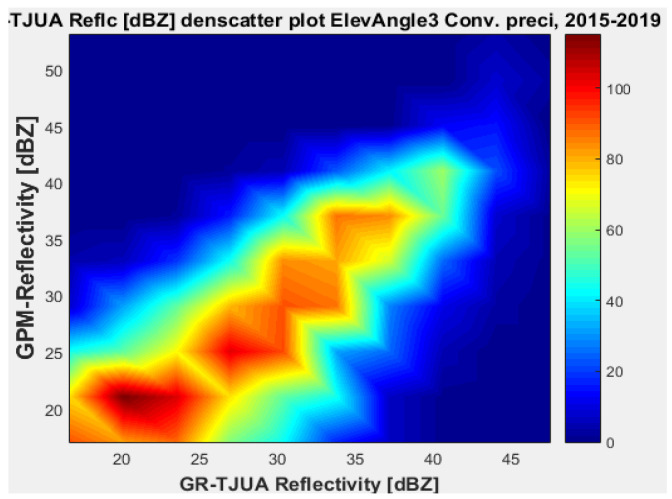
GPM-Ku vs. NEXRAD TJUA for convective precipitation (angle 3).

Figure 15, Figure 16, Figure 17 and Figure 18 represent the scatter density plots for this case for GR angle 3 elevation and the precipitation type.

The composition of the precipitation type for angle 3 is around 37.76% stratiform, 60.81% convective, and approximately 0.14% is classified as other. For GR angle 3, KuPR has better matching for convective and stratiform precipitation, as shown in Figure 16 and Figure 18. Likewise, the variance is lower in KuPR for stratiform precipitation than it is for KaPR; however, for convective precipitation it is the opposite, where KaPR has lower variance.

#### 4.1.4. Angle 4 (3.125°)

**Figure 19:**
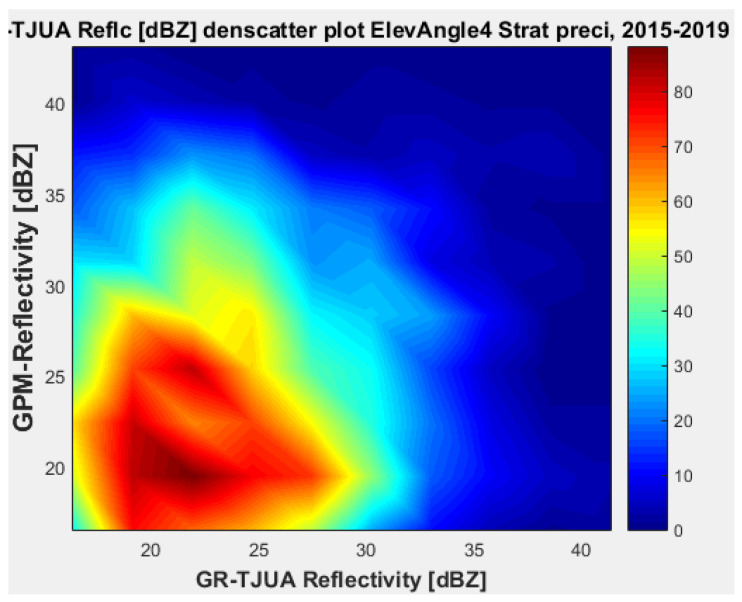
GPM-Ka vs. NEXRAD TJUA for stratiform precipitation (angle 4).

**Figure 20:**
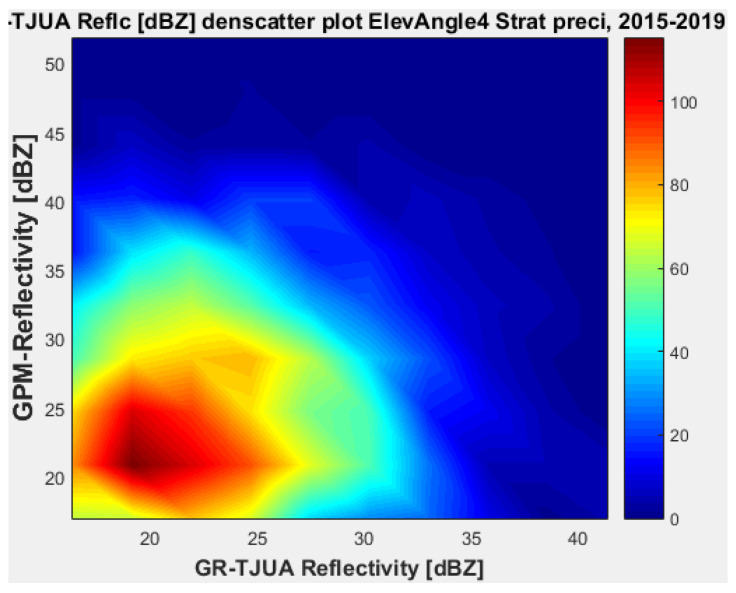
GPM-Ku vs. NEXRAD TJUA for stratiform precipitation (angle 4).

**Figure 21:**
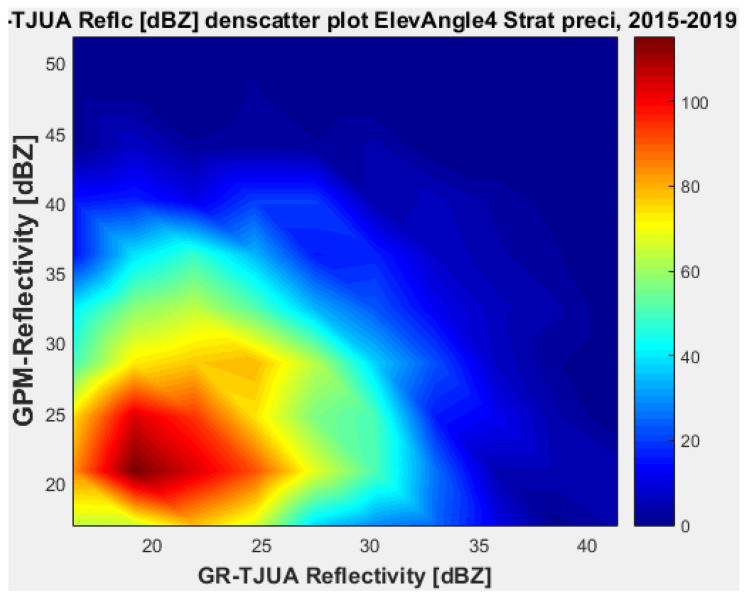
GPM-Ka vs. NEXRAD TJUA for stratiform precipitation (angle 4).

**Figure 22:**
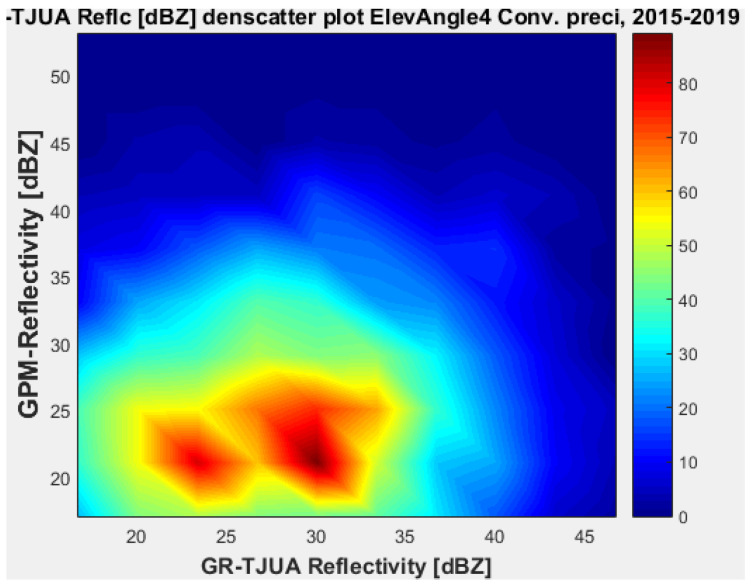
GPM-Ku vs. NEXRAD TJUA for convective precipitation (angle 4).

Figure 19, Figure 20, Figure 21 and Figure 22 represent the scatter density plots for this case for GR angle 4 elevation and the precipitation type.

Finally, for normal weather conditions, the composition of the precipitation type for angle 4 is around 42.54% stratiform, 53.71% convective, and approximately 3.75% classified as other. The mean reflectivity difference from angle 4 shows that KuPR has better correspondence with GR and lower variance than KaPR. For this angle, the stratiform precipitation data are biased to GPM.

### 4.2. Extreme Weather Conditions

This section presents the statistical results of the four extreme weather events, Hurricane Irma, Tropical Strom Beryl, Hurricane Dorian, and Tropical Storm Karen.

#### 4.2.1. Hurricane Irma

Table 3 shows the statistical results for Hurricane Irma comparing the elevations angles and the precipitation type.

For GR angle 1 (0.4843°) during Hurricane Irma, the precipitation type samples are 44.58% stratiform and 55.42% convective, with no precipitation classified as other. Comparing the biases for KuPR and KaPR, they are marginally better for convective precipitation, while the variance for convective is less than half the values obtained for the stratiform types. All precipitation types in angle 1 are also biased toward the GR; in addition, convective type precipitation for Hurricane Irma has the best CC of all the elevation angles.

For GR elevation angle 2 (1.31°) for both Ku and Ka, the precipitation type samples are 41.24% stratiform and 58.76% convective. In terms of the variance, angle 2 shows the same behavior as angle 1, with the convective precipitation type being less than half the values obtained for the stratiform types; in addition, angle 2 is biased toward GR. The bias was better for the stratiform types, with Ka having less bias.

The statistical results for GR elevation angle 3 (2.42°) for both Ku and Ka show that the precipitation type samples are 37.97% stratiform and 62.03% convective, with no precipitation classified as other. In terms of the bias, the behavior of angle 3 is similar to that of angle 2, in which Ka stratiform type has the least bias, followed by Ku convective; however, only Ku convective type has a low variance compared to the other cases. All precipitation types are biased toward GR.

For GR elevation angle 4 (3.125°) for both Ku and Ka, the precipitation type samples are 38.24% stratiform and 61.76% convective, with no precipitation classified as other. In terms of the bias, the behavior is similar to that for angle 3; Ka stratiform type has the least bias followed by Ku convective, with Ku convective type having the lowest variance compared to the other cases. Overall, the bias values are worse for angle 4 than they are for angle 3, and they are also all biased toward GR.

#### 4.2.2. Tropical Storm Beryl

Table 4 presents the statistical results for Tropical Storm Beryl.

For GR angle 1 (0.4843°) during Beryl, the precipitation type samples are 30% stratiform and 67% convective, with 3% classified as other. As in the Hurricane Irma case, the bias for KuPR and KaPR are better for convective precipitation. Likewise, convective type precipitation has better CC than stratiform type, and it is also biased toward the GR [23].

For GR elevation angle 2 (1.31°) for both Ku and Ka, the precipitation type samples are 35% stratiform, 64% convective, and 1% for other types. Considering the bias, angle 2 is biased toward GR. For this angle, the bias was better for the stratiform types, with the bias for Ka being less, similar to the case for Hurricane Irma.

For the results of elevation angle 3 (2.42°), the precipitation type sample distributions are 36% stratiform and 61% convective, with 3% classified as other types. In terms of the bias, angle 3 is similar to angle 2 in which Ka stratiform type has the least bias followed by Ku convective,

For GR elevation angle 4 (3.125°), the precipitation type samples are 34% stratiform, 56% convective, and 8% classified as other, for Ku. For Ka, precipitation type samples are 34% stratiform, 62% convective, and 4% classified as other. In this angle, Ka stratiform type has the least bias, followed by Ku convective; Ku convective type has the lowest variance compared to the other cases.

#### 4.2.3. Hurricane Dorian

Table 5 presents the statistical results for Hurricane Dorian.

For GR angle 1 (0.4843°) during Hurricane Dorian, the precipitation type samples are 39% stratiform and 61% convective, with no precipitation classified as other. The results for this angle are similar to those for Hurricane Irma and Tropical Storm Beryl, in that both precipitation types are biased toward the GR, and the convective type precipitation has a better CC than stratiform type.

Similarly, angle 2 is biased toward GR like angle 1, but the bias was better for the stratiform types. For both Ku and Ka, the precipitation type samples are 44.26% stratiform and 58.76% convective. 

The statistical results of GR elevation angle 3 (2.42°) show that for both Ku and Ka, the precipitation type samples are 37.97% stratiform and 55.73% convective, with no precipitation classified as other. In terms of the bias, Ka stratiform type has the least bias followed by Ku convective; however, only Ku convective type has a low variance compared to the other cases.

For GR elevation angle 4 (3.125°) for both Ku and Ka, the precipitation type samples are 59% stratiform and 41% convective, with no precipitation classified as other. The variance is high for the four angles, but angle 4 presents a lower variance for the convective precipitation. Likewise, the correlation coefficients are low, where KaPR has worse results, especially for angle 4.

#### 4.2.4. Tropical Storm Karen

Table 6 shows the statistical results for Tropical Storm Karen.

Tropical storm Karen is the weakest of the previous extreme events, and unlike the others with similar behaviors for the first three angles, the results obtained for this event are different. The first place for the convective type of KuPR in all four angles is biased to GPM. On the other hand, for stratiform precipitation, angles 1 and 2 of KuPR are biased to GR, while angles 3 and 4 are biased to GPM. Considering the CCs for angles 1 and 2, the CCs are higher for KuPR; however, for angles 3 and 4 the CCs are slightly better for KaPR.

Comparing the results of normal weather cases with the four extreme weather events, there is better correspondence in the results obtained for cases between 2015 and 2019, this in part due to the fact that there are many more samples. According to the statistical analysis and scatter density plots, for normal weather cases the reflectivity difference for every case is biased toward the GR except for angle 4 Ku-band and the stratiform case. Likewise, Ku-band has the best matching in every case for the stratiform and convective cases. For the Hurricane Irma case, the mean reflectivity difference is biased toward the GR (negative bias) for each elevation angle of the GR, and also for each GPM band. The first elevation angles (0.48 and 1.31 degrees) show better matching than the values obtained for angles 3 and 4 (2.42 and 3.125 degrees) in terms of the mean reflectivity difference and variance.

Concerning the precipitation type, convective precipitation shows less variability compared to the stratiform precipitation in Ku-band and Ka-band. For the elevation angle, Ku-band shows substantially less variability in the higher elevation angles when compared to Ka-band. On the other hand, during normal weather conditions, an elevation angle for GR of around 3.39 degrees gives the best matching in terms of bias, variability, and CC; for the case of Hurricane Irma, an elevation angle of 0.48 degrees offers better results.

Of significance is that Hurricane Irma, Tropical Storm Beryl, and Hurricane Dorian showed lower biases and variances for precipitation classified as convective when compared to stratiform for DPR-Ku; likewise, most cases exceeded 5 dBZ and were highly variable except for convective type precipitation. For the cases in 2015–2019, stratiform precipitation generally showed lower values of bias than the convective type.

Regarding the correlation coefficient (CC), for normal weather cases and stratiform precipitation, the CCs for KuPR are between 0.67 and 0.70, and for KaPR they are 0.57–0.70. Likewise, for convective precipitation, the CCs for KuPR are 0.69–0.70 and for KaPR they are 0.67–0.72. These CCs are much lower compared to the results obtained in the study carried out by [20], which quantitatively compared GPM’s observations of reflectivity with instantaneous rainfall products of five NEXRAD ground radars located in the southeastern plains of the U.S.A. Table 7 shows the correlation coefficients obtained by [20] classified into precipitation type.

For the Hurricane Irma case, the CCs for stratiform precipitation are between 0.4765 and 0.5174 for KuPR and between 0.4880 and 0.5807 for KaPR. For convective precipitation, the CCs for KuPR range from 0.4941 to 0.8169 and for KaPR the CCs are 0.4936–0.8200, where the higher values are from angle 1.

The CC range for Tropical Storm Beryl related to stratiform precipitation is between 0.417 and 0.584 for KuPR, and 0.416–0.494 for KaPR. For convective precipitation, the CCs are significantly higher than those for stratiform type, since the CCs for KuPR range from 0.79 to 0.87, while for KaPR they are 0.76–0.86, where the higher values are from angle 1.

On the other hand, Hurricane Dorian exhibited similar behavior to Hurricane Irma. The CCs for stratiform precipitation are between 0.53 and 0.68 for KuPR, and between 0.52 and 0.67 for KaPR; these values are slightly better than those for Hurricane Irma. For convective precipitation, the CCs for KuPR range from 0.40 to 0.55, and for KaPR are from 0.34 to 0.54; they are significantly lower than the corresponding CCs for Hurricane Irma and Tropical Storm Beryl.

Finally, the CCs for Tropical Storm Karen are the greatest of the four extreme weather events for both cases, stratiform and convective precipitation types. The CCs for stratiform precipitation are between 0.86 and 0.90 for KuPR, and between 0.79 and 0.94 for KaPR; these values are slightly better than those corresponding to Hurricane Irma. For convective precipitation, the CCs for KuPR range from 0.87 to 0.93, and for KaPR are from 0.88 to 0.94.

These results indicate that it is necessary to apply corrective algorithms in order to improve the calibration of the GR located in Puerto Rico, and to increase the correlation of the data between GR and GPM. As the event becomes more extreme, the correlation coefficient decreases. Implementing corrective algorithms is a necessary action, considering that the GR is the main instrument used by the government of this country to design forecasts and issue alerts to the community.

## 5. Conclusions

This study performed a cross-evaluation of reflectivity from GPM-DPRs for both Ku- and Ka-band against the ground-based radar NEXRAD located in Puerto Rico (TJUA), for two cases: during normal weather precipitation events and during four extreme weather events.

Data from TJUA in 2015–2019 (normal precipitation cases) and from the extreme weather events were compared in terms of biases and correlation coefficients, and used the first four matching elevation angles for the three scanning modes of the GR, and subsequently categorized by the type of precipitation (stratiform and convective).

The statistical analysis shows that Ku-band possesses the least bias and variability when compared to ground radar reflectivity; for this reason, DPR-Ku is better suited for reflectivity measurements in normal to moderate weather conditions in the Caribbean Region close to Puerto Rico.

Furthermore, the results showed that the elevation angle of the GR has a strong impact in how well the reflectivities match. Likewise, an elevation angle of 3.39 degrees was determined as the best to use for DPR-Ku in normal weather conditions, while for a severe event such as Hurricane Irma, a lower elevation angle such as 0.4843 degrees has the best matching for DPR-Ku and Ka.

The precipitation type also has a significant impact on how well matched the GR and DPR data are. For normal weather precipitation conditions, the stratiform type is statistically better for every GR elevation angle in comparison to the convective type. Similarly, when there are a lower number of convective types samples, the matching is improved, as is the case when the GR elevation angle is higher. Similarly, for Hurricane Irma, Tropical Storm Beryl, and Hurricane Dorian, the precipitation type also had a substantial impact on DPR-GR matching, with a lower GR elevation angle and convective type offering the best match.

However, in terms of the correlation coefficients for both cases, normal weather precipitation conditions and three of the extreme events (Hurricane Irma, Tropical Strom Beryl, and Hurricane Dorian), the results are lower than those from other studies that compared GPM-DPR observations with different NEXRAD locations in the U.S.A; therefore, it is necessary to apply corrective algorithms in order to improve the calibration of the GR located in Puerto Rico. It is necessary to increase the correlation of the data between GR and GPM so that they can provide accurate information for both rain events under normal conditions, and for severe events such as during tropical cyclones.

## Figures and Tables

**Table 1:** Elevation angles and their maximum beam heights.

Angle		Maximum Beam Height (Km)
1	0.48°	0.8377
2	1.31–1.45°	2.53
3	2.42°	4.22
4	3.125–3.39°	5.91

**Table 2:** Statistical results for normal weather conditions.

Normal Weather Conditions					
		KuPR		KaPR	
Angle		Stratiform	Convective	Stratiform	Convective
1	Bias	−0.792329193	−1.928060201	−0.8233397	−2.147966575
	Variance	19.05459973	33.65576683	18.24078643	30.70512149
	MAE	3.198405797	4.698553872	3.122685476	4.571603734
	RMS	4.435640543	6.112789433	4.348742085	5.942434021
	Samples	2548	4816	2563	4828
	CC	0.707132246	0.695508443	0.701784846	0.690715355
2	Bias	−0.643999145	−1.959468715	−0.771038608	−2.230547626
	Variance	19.95418399	32.30120898	19.10399729	29.52500261
	MAE	3.224176468	4.611003214	3.163506815	4.510469111
	RMS	4.512430194	6.01102083	4.437559084	5.873053157
	Samples	2895	3866	2909	3889
	CC	0.686310925	0.72902739	0.677008897	0.724902355
3	Bias	−0.26332424	−1.307805767	−1.672383595	−2.154162215
	Variance	19.48338376	33.30169215	23.58685517	31.67090546
	MAE	3.2547436	4.615509813	4.000603845	4.723886572
	RMS	4.421061512	5.915974923	5.135681587	6.024842945
	Samples	2808	2506	2776	2516
	CC	0.671654104	0.704138687	0.570034514	0.680690284
4	Bias	−0.26332424	−1.307805767	−1.852530874	−2.493749553
	Variance	19.48338376	33.30169215	20.68760321	30.70949712
	MAE	3.2547436	4.615509813	3.842132901	4.788769913
	RMS	4.421061512	5.915974923	4.910250175	6.075513292
	Samples	2808	2506	2320	1870
	CC	0.671654104	0.704138687	0.591936883	0.672371389

**Table 3:** Statistical results for Hurricane Irma.

		KuPR		KaPR	
Angle	Statistics	Stratiform	Convective	Stratiform	Convective
1	Bias	−2.94313838	−2.821463626	−2.952524082	−2.850856449
	Variance	47.79819374	17.01252891	46.04521328	16.73380946
	samples	37	46	37	46
	CC	0.489350541	0.816860184	0.48798682	0.819952168
	MAE	5.571226223	3.859934019	5.591467213	3.824566883
	MSE	55.16841419	24.60334832	53.51814651	24.49741349
	RMS	7.42754429	4.960176239	7.315609784	4.949486184
2	Bias	−2.555253983	−4.662028296	−2.508374166	−4.876060921
	Variance	49.33313154	43.57828099	47.92946176	42.50447784
	Samples	40	57	40	57
	CC	0.491266891	0.632773129	0.490170722	0.616068108
	MAE	5.765949059	5.779512272	5.725718832	6.06207774
	MSE	54.62912617	64.54825758	53.02316618	65.53475535
	RMS	7.391151884	8.034193026	7.281700775	8.095353936
3	Bias	−3.442076715	−4.692876602	−3.55133187	−5.081425375
	Variance	63.05476463	55.53031483	58.85108141	51.8369617
	Samples	30	49	30	49
	CC	0.47654745	0.530448225	0.499089749	0.501101383
	MAE	6.71135931	6.653468521	6.684085687	6.683443537
	MSE	72.80083126	76.4201339	69.50133675	76.59994836
	RMS	8.532340315	8.741861009	8.336746173	8.752139645
4	Bias	−3.487401009	−4.942844187	−4.157013245	−5.503968988
	Variance	52.35617101	49.08833575	44.4351132	41.66927182
	Samples	26	42	25	42
	CC	0.51742935	0.494104255	0.569439427	0.49362148
	MAE	6.236443079	6.576980069	6.208148499	6.529815061
	MSE	62.50443792	72.3512745	59.93846779	70.97082093
	RMS	7.905974824	8.505955237	7.741993786	8.424418136

**Table 4:** Statistical results for Tropical Storm Beryl.

		KuPR		KaPR	
Angle	Statistics	Stratiform	Convective	Stratiform	Convective
1	Bias	−4.188757324	−1.611068541	−4.119752185	−2.023071303
	Variance	29.69152265	27.74995273	27.92358558	27.5691715
	Samples	30	67	30	67
	CC	0.417403818	0.855086534	0.416934835	0.860353451
	MAE	4.995158386	4.309867275	4.804879443	4.595677347
	MSE	46.24749315	29.93131618	43.96515745	31.25050883
	RMS	6.800550944	5.470952036	6.630622705	5.590215455
2	Bias	−3.96698755	−3.094893278	−3.785002589	−3.461226754
	Variance	30.06729915	27.45351222	28.70518371	25.46765433
	Samples	32	59	32	59
	CC	0.44173825	0.82912417	0.432862925	0.82865775
	MAE	4.918266118	4.777979673	4.6668787	5.012819953
	MSE	44.86468627	36.56656286	42.13439132	37.01608981
	RMS	6.698110649	6.047029259	6.491100933	6.084084961
3	Bias	−2.309407976	−3.509221013	−2.604671902	−4.84570752
	Variance	28.40046399	27.28209017	30.59790247	28.18860375
	Samples	27	45	27	46
	CC	0.529551794	0.793626593	0.49425288	0.77078715
	MAE	4.490690726	5.018928274	4.659091243	6.173538332
	MSE	32.68196015	38.99045362	36.24896254	51.05668939
	RMS	5.716813811	6.24423363	6.020711132	7.145396377
4	Bias	−0.975679831	−3.669306331	−1.153117085	−6.505941709
	Variance	24.80327233	11.49199802	32.2660198	15.71218863
	Samples	22	36	20	36
	CC	0.58487078	0.875244005	0.455514283	0.760631905
	MAE	4.100157218	4.201406161	4.469336605	6.817758348
	MSE	24.62780199	24.63658481	31.98239782	57.60301647
	RMS	4.962640627	4.963525441	5.655298208	7.589665109

**Table 5:** Statistical results for Hurricane Dorian.

		KuPR		KaPR	
Angle	Statistics	Stratiform	Convective	Stratiform	Convective
1	Bias	−2.38389152	−4.20767755	−2.304239854	−4.658222198
	Variance	40.15040336	68.38312472	38.81483751	62.83222884
	Samples	23	36	23	36
	CC	0.538710059	0.473353709	0.527199554	0.443717362
	MAE	5.168910773	7.117181645	5.199398704	7.266365634
	MSE	44.08767243	84.18814384	42.43675718	82.7859232
	RMS	6.63985485	9.175409737	6.514350096	9.098677003
2	Bias	−2.28582089	−4.071136222	−2.137696407	−4.642113489
	Variance	30.76966538	57.16153013	30.67199076	50.49311839
	Samples	27	34	27	34
	CC	0.683052459	0.565677187	0.679931993	0.548841922
	MAE	4.381723439	6.167973939	4.437851553	6.237545939
	MSE	34.85502529	72.05445879	34.10573703	70.55724432
	RMS	5.903814469	8.488489783	5.840011732	8.39983597
3	Bias	−0.990524754	−2.85947046	−1.592194641	−3.820850403
	Variance	27.46570967	56.70861986	30.14648051	47.42463277
	Samples	33	31	34	31
	CC	0.614723621	0.552030308	0.538029026	0.5260822
	MAE	3.912260345	5.906849984	4.325223951	5.536262543
	MSE	27.61455473	63.05588085	31.79490309	60.49370371
	RMS	5.254955255	7.940773316	5.638696932	7.777769842
4	Bias	−0.813496431	−1.351946259	−1.90059691	−3.129214325
	Variance	22.36567372	60.37829684	25.97597365	54.17977911
	Samples	36	25	36	25
	CC	0.62558895	0.403635626	0.523370099	0.348124607
	MAE	3.372445424	5.858706818	3.999924898	5.634678345
	MSE	22.40618145	59.79092365	28.86668744	61.80457024
	RMS	4.733516817	7.732459095	5.372772789	7.861588277

**Table 6:** Statistical results for Tropical Storm Karen.

		KuPR		KaPR	
Angle	Statistics	Stratiform	Convective	Stratiform	Convective
1	Bias	−1.5556556	1.706651317	−1.806369029	−0.793639024
	Variance	8.672564753	8.106865581	9.316739678	2.46326481
	samples	71	36	71	36
	CC	0.86673526	0.937141932	0.837512015	0.934651891
	MAE	2.370126778	2.555990166	2.527218563	1.31192202
	MSE	10.9704803	10.79433359	12.44848706	3.024703688
	RMS	3.312171539	3.285473115	3.528241356	1.739167527
2	Bias	−0.602297948	1.480800735	−0.815835025	−0.150544167
	Variance	5.857037297	6.640760467	6.034862038	2.335265974
	Samples	75	36	75	36
	CC	0.903787903	0.930935096	0.876159423	0.946492355
	MAE	1.889265315	2.223025746	1.974323667	1.189369731
	MSE	6.141706285	8.649065714	6.619983998	2.293061021
	RMS	2.478246615	2.940929396	2.572932956	1.514285647
3	Bias	0.801119123	2.871341123	−2.132589764	0.06831736
	Variance	6.242063048	6.562596797	5.661498234	4.379294642
	Samples	84	36	81	36
	CC	0.874454055	0.877279437	0.844826152	0.887496647
	MAE	2.126662118	3.117811468	2.542521371	1.659194893
	MSE	6.809544623	14.62490228	10.1395423	4.26231483
	RMS	2.609510418	3.824251859	3.184264797	2.064537437
4	Bias	2.092964198	2.910401053	−1.220398197	−1.353463411
	Variance	2.919875752	5.280831345	4.885140215	3.443681709
	Samples	75	36	73	36
	CC	0.891335296	0.89085389	0.795730659	0.908970434
	MAE	2.362140477	3.095258633	1.825778844	1.951402744
	MSE	7.261443209	13.60457587	6.307592247	5.17988709
	RMS	2.694706516	3.688438135	2.511492036	2.27593653

**Table 7:** Correlation coefficients for DPR Ku-band and Ka-band reflectivity vs. NEXRAD S-band reflectivity [20].

Nexrad Radar	KuPR		KaPR	
	Stratiform (CC)	Convective (CC)	Stratiform (CC)	Convective (CC)
KFWS (Dallas/Ft. Worth, TX, USA)	0.89	0.88	0.82	0.82
KHGX (Houston/Galveston, TX, USA)	0.88	0.89	0.78	0.83
KSHV (Shreveport, LA, USA)	0.90	0.85	0.82	0.80
KLIX(New Orleans, LA, USA)	0.89	0.84	0.79	0.76
KMLB (Melbourne, FL, USA)	0.83	0.86	0.66	0.71

## References

[1] de Beurs K.M., McThompson N.S., Owsley B.C., Henebry G.M. (2019). Hurricane damage detection on four major Caribbean islands. Remote Sens. Environ..

[2] NSF and The University of Rhode Island (2010). Rainfall and Inland Flooding. http://hurricanescience.org/society/impacts/rainfallandinlandflooding/.

[3] Ortega-Gonzalez L., Acosta-Coll M., Piñeres-Espitia G., Butt S.A. (2021). Communication protocols evaluation for a wireless rainfall monitoring network in an urban area. Heliyon.

[4] Ren Y., Zhang J., Guimond S., Wang X. (2019). Hurricane Boundary Layer Height Relative to Storm Motion from GPS Dropsonde Composites. Atmposphere.

[5] Trepanier J. (2020). North Atlantic Hurricane Winds in Warmer than Normal Seas. Atmposphere.

[6] Yang K., Davidson R.A., Blanton B., Colle B., Dresback K., Kolar R., Nozick L.K., Trivedi J., Wachtendorf T. (2019). Hurricane evacuations in the face of uncertainty: Use of integrated models to support robust, adaptive, and repeated decision-making. Int. J. Disaster Risk Reduct..

[7] Luitel B., Villarini G., Vecchi G. (2018). Verification of the skill of numerical weather prediction models in forecasting rainfall from U.S. landfalling tropical cyclones. J. Hydrol..

[8] Ramirez-Cerpa E., Acosta-Coll M., Velez-Zapata J. (2017). Analysis of the climatic conditions for short-term precipitation in urban areas: A case study Barranquilla, Colombia. Idesia.

[9] Neeck S.P., Kakar R.K., Azarbarzin A.A., Hou A.Y. (2014). Global Precipitation Measurement (GPM) launch, commissioning, and early operations. Sens. Syst. Next-Gener. Satell. XVIII.

[10] Omranian E., Sharif H.O., Tavakoly A.A. (2018). How Well Can Global Precipitation Measurement (GPM) Capture Hurricanes? Case Study: Hurricane Harvey. Remote Sens..

[11] Furukawa K., Yamamoto K., Kubota T., Oki R., Iguchi T. (2018). Current status of the Dual-frequency precipitation Radar on the Global Precipitation Measurement core spacecraft and scan pattern change test operations results. Remote Sens. Atmos. Clouds Precip. VII.

[12] Hou A.Y., Kakar R.K., Neeck S., Azarbarzin A.A., Kummerow C.D., Kojima M., Oki R., Nakamura K., Iguchi T. (2014). The Global Precipitation Measurement Mission.

[13] Skofronick-Jackson G., Petersen W.A., Berg W., Kidd C., Stocker E.F., Kirschbaum D.B., Kakar R., Braun S.A., Huffman G.J., Iguchi T. (2017). The Global Precipitation Measurement (GPM) Mission for Science and Society. Bull. Am. Meteorol. Soc..

[14] Baldini L., Roberto N., Montopoli M., Adirosi E. (2018). Ground-Based Weather Radar to Investigate Thunderstorms. Remote Sensing of Clouds and Precipitation.

[15] Acosta-Coll M., Ballester-Merelo F., de la Hoz-Franco E., Martinez-Peiró M. (2018). Real-time early warning system design for pluvial flash floods—A review. Sensors.

[16] Keem M., Seo B.C., Krajewski W.F., Morris K.R. (2019). Intercomparison of Reflectivity Measurements between GPM DPR and NEXRAD Radars. Atmos. Res..

[17] Biswas S., Chandrasekar V. (2018). Cross-Validation of Observations between the GPM Dual-Frequency Precipitation Radar and Ground Based Dual-Polarization Radars. Remote Sens..

[18] Kim K., Bui L. (2019). Learning from Hurricane Maria: Island ports and supply chain resilience. Int. J. Disaster Risk Reduct..

[19] López-Marrero T., Castro-Rivera A. (2019). Let’s not forget about non-land-falling cyclones: Tendencies and impacts in Puerto Rico. Nat. Hazards.

[20] Bacopoulos P. (2019). Extreme low and high waters due to a large and powerful tropical cyclone: Hurricane Irma (2017). Nat. Hazards.

[21] Benach J., Diaz M.R., Muñoz N.J., Martinez-Herera E., Pericas J.M. (2019). What the Puerto Rican hurricanes make visible: Chronicle of a public health disaster foretold. Soc. Sci. Med..

[22] Colom J.G., Cruz-Pol S., Pablos G., Córdoba M.F., Castellanos W., Acosta M., Ortiz J.A., de Jesús B., Trabal J. (2010). Uprm Weather Radars at the Central American and Caribbean Games at Mayagüez 2010. IEEE Geosci. Remote Sens. Lett..

[23] Zhong L., Yang R., Wen Y., Chen L., Gou Y., Li R., Zhou Q., Hong Y. (2017). Cross-evaluation of reflectivity from the space-borne precipitation radar and multi-type ground-based weather radar network in China. Atmos. Res..

[24] Morris K.R., Greenbelt S., Schwaller M.R. Sensitivity of Spaceborne and Ground Radar Comparison Results to Data Analysis Methods and Constraints. Proceedings of the 35th Conference on Radar Meteorology.

[25] Biswas S.K., Chandrasekar V. Cross validation of observations from GPM dual-frequnecy precipitation radar with S-band ground radar measurents over the Dallas—Fort worth region. Proceedings of the 2017 IEEE International Geoscience and Remote Sensing Symposium (IGARSS).

[26] Arias I., Chandrasekar V. Cross Validation of GPM and Ground-Based Radar in Latin America and the Caribbean. Proceedings of the IGARSS 2018-2018 IEEE International Geoscience and Remote Sensing Symposium.

[27] Goddard Space Flight Center (2013). Global Precipitation Mission (GPM) Ground Validation System Validation Network Data Product User’s Guide. https://gpm.nasa.gov/sites/default/files/document_files/Val_Network_Users_Guide_v4.1.pdf.

[28] National Hurricane Center Hurrican Beryl. https://www.nhc.noaa.gov/data/tcr/AL022018_Beryl.pdf.

[29] National Weather Service (2019). Hurricane Dorian. https://www.weather.gov/mhx/Dorian2019.

[30] National Weather Service (2019). Tropical Storm Karen. https://www.weather.gov/sju/karen2019.

